# Comparing Latin American nutrient profile models using data from packaged foods with child-directed marketing within the Brazilian food supply

**DOI:** 10.3389/fnut.2022.920710

**Published:** 2022-12-02

**Authors:** Camila Aparecida Borges, Neha Khandpur, Daniela Neri, Ana Clara Duran

**Affiliations:** ^1^Center for Epidemiological Research in Nutrition and Health, Faculty of Public Health, University of São Paulo, São Paulo, Brazil; ^2^Center for Food Studies and Research, State University of Campinas, São Paulo, Brazil; ^3^Department of Nutrition, School of Public Health, Harvard University, Boston, MA, United States

**Keywords:** nutrient profile model, food labeling, food policies, child-directed marketing, marketing, marketing to children, unhealthy food marketing

## Abstract

**Objectives:**

This study aimed to examine and compare the extent to which different nutrient profile models (NPMs) from Latin America (LA) identify packaged foods and beverages with child-directed marketing sold in Brazil as being high in nutrients associated to the risk of non-communicable diseases (NCDs).

**Materials and methods:**

In this cross-sectional study, we evaluated 3,464 foods found in the five largest Brazilian supermarkets. Child-directed marketing was coded using the International Network for Food and Obesity/NCDs Research, Monitoring and Action Support (INFORMAS) protocol. Differences in medians of sugar, saturated fats, and sodium per 100 kcal in foods, with the presence and absence of child-directed marketing, were tested using the Mann–Whitney test. We compared six NPMs in LA and examined to what extent they targeted these products using prevalence ratios. Analyses were performed overall and by the degree of food processing according to the Nova food classification.

**Results:**

We found 1,054 packages with child-directed marketing. Among these, candies, cakes and pies, sauces and creams, and sugar-sweetened beverages were significantly higher in sugar, saturated fat, and sodium per 100 kcal than products that are not targeted at children (*p* < 0.05). Compared with PAHO and the Mexico models, the Brazilian NPMs would allow three times more ultra-processed foods to omit warnings for sodium (*p* < 0.05). The Uruguayan NPM also flagged fewer ultra-processed foods high in sodium (*p* < 0.05). The Brazilian model also allows four times more sugar-sweetened beverages and six times more dairy drinks to omit warnings for sugar than the Mexico and PAHO models. In comparison to all other NPMs, the Brazilian model showed the worst performance in identifying baked goods as high in sodium. Chile, Uruguay, and Peru models would also target significantly less sugar-sweetened beverages and high in at least one critical nutrient than PAHO and Mexico models.

**Conclusion:**

Compared with other NPMs in LA, the NPM criteria adopted in Brazil are more permissive and less likely to inform consumers of the poor nutritional quality of ultra-processed foods and beverages with child-directed marketing.

## Introduction

Childhood obesity has become a global epidemic and carries significant long-term consequences to physical and mental health (1), including an increased risk of the development of diet-related chronic diseases and worse psychological health and socioeconomic outcomes (2, 3). Moreover, excessive weight gain in childhood increases the risk of being overweight and obese in adulthood (4). According to the World Health Organization (WHO), in 2019, an estimated 38.2 million children were overweight or obese worldwide (5). The prevalence of obesity among children and adolescents (ages 2–19 years) in Latin America (LA) is among the highest in the world, with one in five individuals either overweight or obese (6).

Ultra-processed food consumption (7–10) and exposure to the marketing of unhealthy foods and beverages (11–16) are linked to growing overweight and obese in childhood. Ultra-processed foods are defined as formulations of ingredients, mostly of industrial use, which results from a series of industrial processes (hence “ultra-processed”) with added sugars, salt, fat, and additives. Some examples of these foods are salty snacks, sugar-sweetened beverages (SSBs), biscuits, candies, and breakfast cereals (17).

Persuasive marketing strategies influence children's and adolescents' food intake, preferences, attitudes, and eating behavior (18–20). They are particularly harmful at this stage of life because their cognitive development is relatively limited, making it harder for them to recognize the persuasive intent of marketing used by the food industry (21, 22). Food marketing may also affect children's purchasing preferences for foods and influence long-term norms related to food consumption (23, 24). Despite that marketing on food packaging is less studied than televised food marketing in addressing childhood obesity (19, 25), current evidence suggests that cartoon characters and other endorsers including brand mascots, celebrities, sports figures comprise the most prevalent type of marketing targeted at children on food packages (19, 22). As the sales of packaged foods in LA rise, particularly that of ultra-processed foods (26), this evidence gap may constrain effective policymaking to prevent childhood overweight and obesity.

Some LA countries have implemented strategies and policies to curb rising obesity rates including taxation of SSBs; front-of-package (FoP) nutritional labeling for nutrients like sugar, saturated fat, and sodium; and marketing and food procurement restrictions (27–30). Evidence from Chile, where a combination of these policies was implemented in 2016, showed that the volume of SSB purchases with FoP nutritional labeling decreased by 22.8 ml/capita/day post regulation (31). Other studies from Chile showed a decrease in hours of children's exposure to TV programs with advertisements using cartoon characters for foods high in energy, saturated fats, added sugars, or sodium (32), and a decline in the proportion of advertisements for foods high in these nutrients of concern after the restrictions were implemented. The sharpest declines were seen for carbonated beverages, desserts, breakfast cereals, and fruit-flavored drinks (33).

Nutritional labeling regulations have recently been revised in Brazil, resulting in new FoP nutritional labeling policies to assist with consumers' food purchase decisions (34). Like in Chile, the updated Brazilian labeling regulation includes a nutrient profile model (NPM) to identify excessive amounts of sugar, saturated fat, and sodium (35, 36). NPMs set eligibility criteria and nutrient thresholds to determine which foods and beverages should be targeted by food policies (28, 30, 37). The NPM adopted in Brazil differs from the models used in other LA countries, including the one endorsed by the Pan American Health Organization (PAHO) (38, 39). The Brazilian model has been reported to be more permissive in limiting nutrients of concern, which could lead to a lower proportion of foods and beverages receiving FoP labels, even though they may have high contents of sodium, saturated fat, and sugar as identified by using other NPMs (39).

Nutrient profile models can also be used to target foods that should have marketing restrictions for children (28, 33, 37, 40–42). Based on the key role that NPMs play in flagging unhealthy products that could face marketing restrictions, the aim of this study is to examine and compare the extent to which different NPMs from Latin America (LA) identify packaged foods and beverages with child-directed marketing sold in Brazil as being high in nutrients associated to the risk of non-communicable diseases (NCDs).

## Materials and methods

### Brazilian food labeling database

In this cross-sectional study, we used data from a sample of packaged foods sold in five Brazilian food retailers with the largest market share in the country (43). The five retailers account for 70% of the sales of branded products, who were identified using the annual food retail sales report generated by Euromonitor International in 2016 (44). Supermarkets were selected as the source of data collection because they account for a large share (59%) of the energy consumed by Brazilians (45). São Paulo, located in the southeast region of the country, was chosen as the primary study area because it is the largest city in Brazil (46). As one of the food retail chains only had stores in the northeast region of the country, data collection at this chain was conducted in Salvador, their largest market.

Data on the geographic location of the five retail chains in São Paulo and Salvador in Brazil were collected from the websites of each company, and the addresses were geocoded. The neighborhood of each store was defined as a 1 km buffer (using Euclidean distance) around the store. The stores were stratified by tertiles of neighborhood income. Information on income from the household top earner was obtained from the 2010 Brazilian Census (46). The largest store of each retail chain in the first and third tertiles was selected to ensure socioeconomic representativeness in the sample, except for one chain that only allowed data collection in its distribution center, where all products sold in the chain were available. Formal permission was obtained from all the supermarkets included in this study.

The sampling of the five food retailers and 128 food groups investigated was based on the sampling recommendations of the International Network for Food and Obesity/Non-communicable Diseases (NCDs) Research, Monitoring and Action Support (INFORMAS) (48). Details about the food groups are available in Supplementary Table 1. The INFORMAS sampling approach was deemed appropriate for this study when factors like potential researcher burden, time, and data collection costs were considered. The approach focuses on food categories clearly related to reducing or increasing the rates of obesity and diet-related NCDs (relevant to INFORMAS objectives), for example, fruits and vegetable products (canned, frozen, etc.).

**Figure 1:**
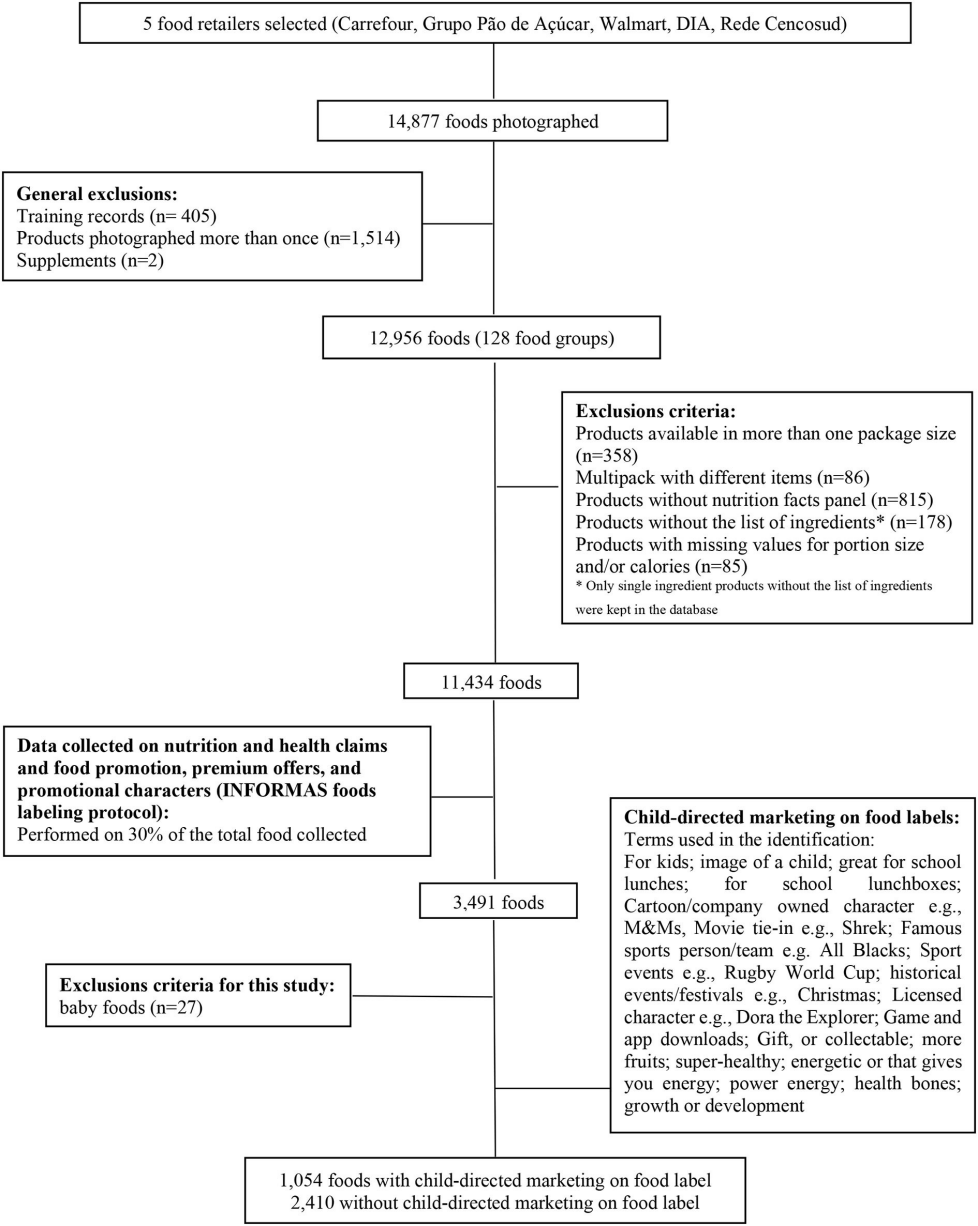
Flowchart of data collection and exclusion process.

Data were collected between April and July 2017 by trained fieldworkers using previously employed protocols (47). Packaged foods and beverages were included, and ~13,000 different items distributed in 128 categories of food products had all sides of their package photographed. Information on the product brand and flavor, package size, nutrition facts panel, ingredients list, and reconstitution instructions, when applied, was entered between July and November 2017 by trained nutritionists. For data on composition information (nutrition facts panel, ingredient list), 10% of the sample was double-entered by the same person and 10% of the sample was repeated by a second person for intra- and inter-rater reliability analyses. After the exclusion of items available in more than one package size, products without nutrition information, multipacks with different items, products without a list of ingredients, and products with missing values for portion size and/or calories, 11,434 records were maintained in the database (Figure 1). More details about the procedures for data collection can be found in a previous study (39).

### Identifying marketing strategies

Data related to marketing strategies were collected from the food labels in a random subsample of the aforementioned packaged foods and beverages. Marketing strategies included nutrition and health claims, food promotion, premium offers, and promotional characters. These data were originally collected to estimate the prevalence of packaged foods and beverages containing marketing strategies in the Brazilian food supply and to estimate the proportion of products that would receive FoP nutritional labeling under the approved Brazilian food labeling regulation.

We used the information on marketing strategies from this random subsample of 30% of all the 128 categories of food products (11,434 foods and beverages), resulting in 3,491 products. We did not find statistically significant differences in food composition when we compared this random sample with the universe of photographed food packages (Figure 1). For this stage of data collection, four researchers with a degree in nutrition science were trained according to protocols developed by INFORMAS (48). The “*Food Labeling Module”* protocol was used, which aims to monitor health-related labeling components and promotional characters and premium offers on packaged foods and non-alcoholic beverages sold in major food outlets (Figure 1). It provides guidance for the data collection on ingredient lists, nutrient declarations, and nutrition and health claims to monitor the components of food labeling offered in food retail outlets in different countries (48). The monitoring of labeling on packaged foods proposed in the protocol captures the presence/absence and other aspects of the list of ingredients (e.g., whether quantitative or not), nutrient declarations, supplementary nutrition information, and all claims (nutrition claims, health claims, and other claims), except for other non-health-related labeling information, for example, date marking and country of origin. Premium offers and promotional characters on food packages are also considered in this protocol (48).

Trained research assistants evaluated the images of all sides of the packages. They identified the presence of claims and categorized the types of terms and content of food labels that could be classified as health-related claims, promotional characters, and premium offers using the taxonomy provided by the INFORMAS protocol (Supplementary Table 2). All visible text on the label could be coded as a claim, including brand names and slogans. For this study, we only considered claims on the front of the package. We neither considered the mention of substances in the list of ingredients as a claim nor considered the mention of nutrients as a mandatory part of nutrition labeling in Brazil.

Data entry was performed between April and June 2017 in Epi Info™ version 7. Baby foods were excluded (*n* = 27 items) because these are regulated by the Brazilian Norm for the Commercialization of Foods for Infants and Young Children, Bottles, Pacifiers, and Breast Protectors (NBCAL), which restricts child-directed marketing (49). Our final sample consisted of 3,464 foods and beverages (Figure 1).

### Identifying child-directed marketing on food labels

Any content shown in all visible text of the label that could be characterized as child-directed marketing was assessed. A report made by the WHO (50) highlights the importance of considering in the definition of what is “child-directed marketing” the unintentional exposure of children, for example, in marketing “directed at adults.” Even if children are not the primary target audience, this exposure can have negative health impacts. In this study, we sought to cover both marketing strategies directed to children and those that can persuade children and their parents or caregivers to purchase and/or consume the product.

To help decide what constitutes child-directed marketing, we combined three categories proposed by INFORMAS' *Food Labeling Module* along with insights from the scientific literature. A search for the scientific literature was conducted using the following terms: childhood/infant/child/adolescent obesity or overweight; marketing; unhealthy foods; child-target; child-directed appeals; child-appeal advertising; child-directed marketing strategies; child-directed marketing; food marketing; advertising to children. From the literature (12–14, 18–22, 30, 51, 52), we verified the following terms and elements: cartoons and mascots, images of children, images of fruits and vegetables, sports and athletes, school elements, growth and development references, energy references, “healthy bones” “super healthy,” among others claims (Supplementary Table 1). These elements, along with health and nutrition claims, promotional characters, and prizes, helped identify a total of 1,054 foods with at least one child-directed marketing strategy on their packages. Details of the variables and terms used to characterize child-directed marketing from the INFORMAS codebook are provided in Supplementary Table 1.

### Nutrient profile models

Given the importance of NPMs in the implementation of further government policies regulating unhealthy food marketing to children (30, 33, 37), we compared the Brazilian NPM with five NPMs proposed or adopted in the LA context. We chose NPMs that are relevant to FoP nutritional labeling and restrictions to unhealthy food marketing to children in LA, given the similarities in the epidemiological, nutritional, economic, and demographic contexts of the region (35, 53). The six NPMs were as follows:

1. NPMs proposed in Brazil (34),
2. NPMs proposed by the Pan-American Healthy Organization (PAHO) (35),
3. NPMs proposed in Chile (36),
4. NPMs proposed in Mexico (54),
5. NPMs proposed in Uruguay (55), and
6. NPMs proposed in Peru (56).

Table 1 shows in detail the characteristics of each of these NPMs, including nutrient thresholds for solids (in grams (g)) and liquids (in milliliters (ml)) and the eligibility criteria. Targeted nutrients of concern differ across NPMs and include energy, saturated fat, total fat, trans fat, sodium, free sugars, total sugar, caffeine, and/or non-nutritive sweeteners. In Brazil, all packaged foods and beverages are eligible to receive an FoP nutritional label (or warning labels), except for fresh fruits and vegetables, tubers, cereals, nuts, seeds and mushrooms, flours, meat, fish and eggs, fermented milk without added sugar, cheese, fluid milk, powdered milk, olive oil and vegetable oils, salt, infant formulas, enteral formulas, weight control foods, supplements, and alcoholic beverages (34). Since the nutrient criteria may differ among the NPMs evaluated, we analyzed and compared those in common among all of them (free sugar, saturated fat, and sodium).

**Table 1:** Characteristics of the six nutrient profiling models used in this study.

**Nutrient profiling models**	**Nutrients of concern**	**Eligibility**
Brazil	Sugar (free sugars): ≥15 g/100 g; ≥7.5 g/100 m Sodium: ≥600 mg/100 g; ≥300 mg/100 ml Saturated fat: ≥6 g/100 g; ≥3 g/100 ml	All foods and beverages. Except: fresh fruits and vegetables; tubers; cereals; nuts and seeds; mushrooms; flours; meat; fish and eggs; fermented milk without added sugar; cheese; fluid milk; powdered milk; vegetable oils; salt; infant formulas; enteral formulas; food for weight control diets; nutritional supplements; alcoholic beverages
PAHO	Sugar (free sugars): ≥10% of total energy Sodium: ≥1 mg per 1 kcal Saturated fat: ≥10% of total energy Trans fat: ≥1% of total energy Presence of non-nutritive sweeteners	Processed and ultra- processed foods and beverage, according to NOVA food classification
Chile (phase three)	Sugar (total sugar): ≥10 /100 g; ≥5 g/100 ml Sodium: ≥400 mg/100 g; ≥100 mg/100 ml Saturated fat: ≥4 g/100 g; ≥3 g/100 ml Energies: ≥1,150 kJ (275 kcal); ≥233 kJ (70 kcal)	Food and beverages with added sugar, sodium, or fat
Mexico (phase three)	Sugar (free sugars): ≥10% of total energy Sodium: ≥1 mg of sodium per 1 kcal or ≥300 mg; ≥45 mg for non-caloric beverages Saturated fat: ≥10% of total energy Trans fat: ≥1% of total energy Energies: ≥275 kcal/100 g; ≥70 kcal/100 ml or ≥8 kcal/100 ml from free sugars Presence of added caffeine Presence of non-nutritive sweeteners	Foods and beverages with free sugars, fat, or sodium
Uruguay	Sugar (free sugars): ≥13 g/100 g; ≥3 g/100 ml or 5 g/100 ml for products without other sweeteners; ≥7 g/100 ml in products with up to 80% of total calories from sugars and no added other sweeteners Sodium: ≥500 mg/100 g; ≥200 mg/100 ml Saturated fat: ≥6 g/100 g; ≥3 g/100 ml Total fat: ≥13 g/100 g; ≥4 g/100 ml	All food and beverages. Except: enteral nutrition; foods for weight control diets; nutritional supplements; infant formulas up to 36 months and table-top sweeteners.
Peru (phase two)	Sugar (total sugar): ≥10 g/100 g; ≥5 g/100 ml Sodium: ≥400 mg/100 g; ≥100 mg/100 ml Saturated fat: ≥4 g/100 g; ≥3 g/100 ml Presence of trans fat	All processed and ultra-processed foods that exceed the targeted nutrients of concern

The term “free sugars” was adopted in this article to refer to both free and added sugars (57). Although Chile and Peru consider total sugar in their NPM, all the sugar present in the foods analyzed was interpreted as free sugars since the labels of foods and beverages sold in Brazil do not have on the nutrition facts panel the information on total sugar or added sugar, but include only carbohydrates. We estimated the content of free sugars using a validated and curated eight-step protocol that uses information available on the list of ingredients and on the carbohydrate content of packaged foods and beverages displayed on the nutrition facts panel (57). This method uses information readily available on most food labels and allows for the estimation of the added sugar content of packaged foods and beverages in countries where both added and total sugar information are not mandated on food labels, which is the case in Brazil (57). These eight-step included objective and subjective estimation procedures for different food groups. Intrinsic sugar found in milk and 100% fruit juices was excluded from the estimates. In addition, one of the steps uses the total sugar content of the product when producers voluntarily make it available in the nutrition facts panel (57).

### Food classification

Food and beverages were classified by the degree of processing using the Nova classification (17), a system that divides foods and beverages into four groups according to the extent and purpose of the industrial processing they undergo. It considers all physical, biological, and chemical methods used during the food manufacturing process, including the use of food additives (17). Nova group 1 includes *unprocessed foods* (composed of fresh fruits and vegetables, and eggs) and *minimally processed foods* (composed of frozen meat, frozen fish, 100% fruit juice, coffee powder, tea herbs, dried cereals and pulses, cocoa powder, plain yogurts, fluid or powdered milk, frozen vegetables, and dried herbs). The remaining three Nova food groups include *culinary ingredients* (sugar, honey, olive oils, oils, cooking fats, salt, and vinegars), *processed foods* (bread, jerky, bacon, canned and dried fish, cheese, canned and dried fruit, and vegetables), and *ultra-processed foods* (soft drinks, sugar-sweetened beverages, dairy drinks, baked goods, breakfast cereals, salty snacks, candies, cakes and pies, dairy desserts, ultra-processed meat, ready-to-eat food, and sauces and creams).

### Statistical analyses

First, we defined whether a food or beverage had any child-directed marketing. We observed the presence of caffeine and non-nutritive sweeteners using the list of ingredients available on food packages. The following components of the list of ingredients were identified as non-nutritive sweeteners: sorbitol, mannitol, acesulfame, aspartame, cyclamate, isomaltose, saccharin, sucralose, thaumatin, stevia, neotame, maltitol, lactitol, xylitol, and erythritol. We then calculated the proportion of foods and beverages with child-directed marketing. Third, using the nutrition information collected from the nutrition facts panel of the products, we calculated the content of free sugars (g), saturated fat (g), and sodium (mg) per 100 Kcal. To classify a food or beverage as high in any nutrient of concern, we used the cutoff points and eligibility criteria defined in each of the NPMs studied (Table 1).

For the descriptive analyses, we first tested the normality of the continuous variables (free sugar, sodium, and saturated fat) through the Shapiro–Wilk test. Once non-normal distributions were confirmed, we then performed the Mann–Whitney test to assess differences in the median of free sugar, sodium, and saturated fat per 100 kcal of the foods and beverages with the presence and absence of child-directed advertising, with significance levels set at a *p*-value of <0.05. The variables were expressed through descriptive statistics using medians and interquartile ranges (p25; p75).

To estimate the proportion of foods with child-directed marketing that should receive at least one FoP nutritional labeling, we used all the nutrients of concern adopted in each NPM. We also estimated the proportion of foods with child-directed marketing that should receive FoP nutritional labeling for free sugar, sodium, and saturated fat, separately. We compared the differences in the proportion of foods and beverages labeled as high in critical nutrients using prevalence ratios and the 95% confidence interval (when the confidence interval of the prevalence ratio did not pass 1, the differences were considered statistically significant). Comparisons were made between Brazil and other models, Mexico and other models, and PAHO and other models, analyses were performed overall and by Nova food groups. All analyses were run in Stata SE version 16.

## Results

**Figure 2:**
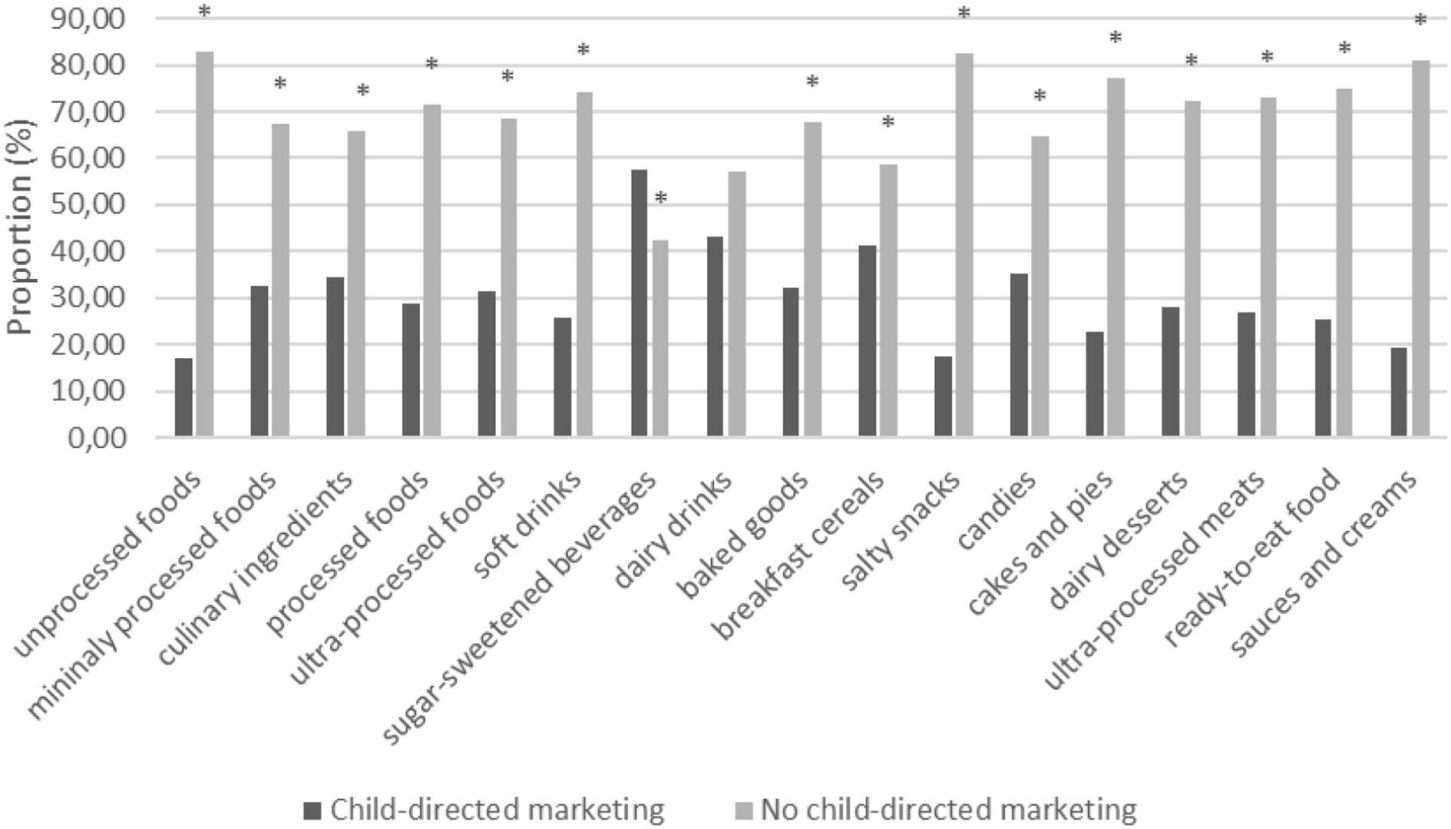
Proportion of Brazilian packaged foods and beverages with the presence or absence of child-directed marketing sold in Brazilian top five food retailers, 2017.

From a total of 3,464 foods and beverages, 1,054 (30%) had at least one child-directed marketing strategy. The remaining 70% of the foods and beverages had other types of claims and marketing strategies. Most with child-directed marketing were classified as ultra-processed foods (n=654, 61%). The sugar-sweetened beverages subgroup had significantly higher proportions of child-directed marketing, 57.5% (95% CI: 50.8–64.0), than other subgroups of ultra-processed foods. We found differences in the proportion of foods with and without child-directed marketing in all food subgroups, except for dairy beverages (Figure 2).

Table 2 shows the comparison of nutrients (free sugars, saturated fat, and sodium) according to the presence or absence of child-directed marketing. Among products with the presence of child-directed marketing, we found higher concentrations of free sugars (g per 100 kcal) in candies (median [IQR] 33.0 g [20.4; 83.0]) (*p* < 0.05), higher concentrations of saturated fat (g per 100 kcal) in cakes and pies (median [IQR] 11.1 g [8.8; 14.0 g]) (*p* < 0.001) and in sauces and creams (median [IQR] 13.7 g [0.0; 42.2 g]) (*p* < 0.05), and higher concentrations of sodium (mg per 100 kcal) in the sugar-sweetened beverages (median [IQR] 32.6 mg [9.1; 161.1 mg]) (*p* < 0.05) and in candies (median [IQR] 37.8 mg [14.5; 76.3 mg]) (*p* < 0.001).

**Table 2:** Median and interquartile range for the content of free sugars, saturated fat, and sodium per 100 kcal in processed and ultra-processed foods by the presence and absence of child-directed marketing (Brazil, 2017).

**Food groups**	**Free sugars (g)**				**Saturated Fat (g)**				**Sodium (mg)**			
	**Child-direct marketing**				**Child-direct marketing**				**Child-direct marketing**			
	**No**		**Yes**		**No**		**Yes**		**No**		**Yes**	
	***n***	**Median (IQR)**	***n***	**Median (IQR)**	***n***	**Median (IQR)**	***n***	**Median (IQR)**	***n***	**Median (IQR)**	***n***	**Median (IQR)**
Processed foods	227	0.0 (0.0; 11.11)	94	0.0 (0.0; 0.0)	226	7.5 (0.0; 39.1)	94	39.4 (12.0; 48.5)	227	247.8 (115.0; 520.0)	94	188.4 (112.0; 294.1)
Ultra-processed foods (total)	1,429	9.9 (0.6; 29.9)	657	8.1 (0.5; 32.2)	1,425	9.4 (0.0; 19.5)	653	5.7 (0.0; 16.1)	1,424	97.34 (25.2; 318.4)	654	70.5 (20.6; 205.9)
Soft drinks	26	12.5 (7.8; 13.3)	9	0.0 (0.0; 0.0)	26	0.0 (0.0; 0.0)	9	0.0 (0.0; 0.0)	26	25.3 (13.8; 50.0)	9	1.1 (1.1; 2.3)
Sugar-sweetened beverages	91	3.1 (2.7; 12.5)	123	3.1 (0.6; 10.6)	91	0.0 (0.0; 0.0)	123	0.0 (0.0; 0.0)	91	16.2 (0.0; 54.5)	123	32.6 (9.1; 161.1)^a^
Dairy drinks	57	6.28 (3.2; 11.9)	43	5.19 (3.2; 9.6)	57	7.0 (3.1; 11.2)	43	7.0 (0.0; 12.8)	57	66.7 (40.0; 156.2)	43	48.5 (8.2; 69.9)
Baked goods	65	5.3 (3.1; 25.8)	31	5.8 (3.1; 35.6)	65	2.2 (0.0; 3.9)	31	3.1 (1.4; 8.5)^a^	65	153.3 (126.2; 185.3)	31	157.7 (127.1; 190.4)
Breakfast cereals	47	33.0 (14.3; 56.6)	33	34.2 (24.2; 58.6)	47	7.2 (1.9; 11.6)	33	7.4 (2.3; 13.7)	47	25.5 (10.0; 47.5)	33	33.7 (16.4; 54.4)
Salty snacks	149	9.4 (1.65; 53.3)	33	7.4 (0.0; 45.8)	147	9.8 (2.9; 18.0)	32	7.9 (3.2; 19.3)	148	101.1 (24.6; 165.8)	33	114.4 (43.8; 144.5)
Candies	262	28.1 (23.7; 36.4)	144	33.0 (20.4; 83.0)^a^	262	15.6 (5.7; 26.5)	143	8.9 (0.0; 21.6)	260	29.6 (11.1; 56.5)	143	37.8 (14.5; 76.3)^a^
Cakes and pies	54	21.9 (16.8; 27.8)	16	23.2 (17.7; 28.9)	54	7.3 (4.5; 10.8)	16	11.1 (8.8; 14.0)^a^	54	81.7 (51.8; 115.6)	16	60.6 (53.6; 72.0)
Dairy desserts	106	19.3 (15.9; 50.7)	41	19.0 (11.3; 36.4)	105	15.3 (2.4; 23.7)	40	9.0 (0.0; 16.1)	105	23.1 (11.8; 33.6)	41	29.7 (4.0; 55.5)
Ultra-processed meat	154	0.3 (0.0; 0.8)	57	0.1 (0.0; 1.2)	153	22.5 (15.0; 30.0)	56	20.8 (14.7; 26.7)	153	442.4 (278.9; 571.4)	56	234.2 (66.1; 430.1)
Ready-to-eat food	187	0.3 (0.0; 3.3)	63	0.6 (0.1; 11.2)	187	11.2 (4.5; 16.1)	63	9.5 (2.8; 16.3)	187	261.1 (189.6; 380.1)	62	239.3 (143.3; 383.6)
Sauces and creams	230	3.2 (0.0; 55.8)	56	2.4 (0.0; 16.5)	230	0.0 (0.0; 14.3)	56	13.7 (0.0; 42.2)^a^	230	773.5 (237.4; 2,030.4)	56	392.4 (171.0; 1108.6)

a Median values significantly higher for free sugars (g), saturated fat (g), and sodium (mg) by the presence of child-directed marketing. Differences in medians was observed by the Mann–Whitney test (p < 0.05).

Table 3 shows the proportion of foods with child-directed marketing that contain a high content of at least one of the nutrients in each NPM. A total of 87% of foods with child-directed marketing were high in at least one nutrient when the PAHO and Mexican NPMs were applied, whereas, only 54% when the Brazilian NPM was applied. Compared with the PAHO NPM, the Brazilian NPM allowed eight times more soft drinks and four times more sugar-sweetened beverages and dairy drinks targeted at children to omit warnings on at least one critical nutrient (*p* < 0.05); when compared to the Mexican NPM, the Brazilian NPM allowed 2.6 times more processed foods, eight times more carbonated soft drinks, 4.7 times more sugar-sweetened beverages, 4.3 times more dairy drinks, and two times more ready-to-eat foods and baked goods to omit warnings on at least one critical nutrient (*p* < 0.05). Compared with the PAHO and Mexican models, the NPMs employed by Chile, Uruguay, and Peru also omitted significantly more warnings on at least one critical nutrient in soft drinks and sugar-sweetened beverages (*p* < 0.05).

**Table 3:** Proportion (%) of foods and beverages with child-directed marketing and a high content of at least one nutrient of concern according to different nutrient profile models (NPMs), overall, and by food category (Brazil, 2017).

	**PAHO**	**Brazil**	**Chile**	**Mexico**	**Uruguay**	**Peru**
**Food groups**	**% (95% CI)**	**% (95% CI)**	**% (95% CI)**	**% (95% CI)**	**% (95% CI)**	**% (95% CI)**
Unprocessed and minimally processed foods	0.00	0.00	0.00	0.00	0.00	0.00
Culinary ingredients	0.00	82.35 (57.23; 94.21)	0.00	6.25 (2.03; 17.70)	75.00 (60.93; 85.23)^b^	83.33 (70.04; 91.45)
Processed foods	91.30 (83.55; 95.60)	35.00 (17.66; 57.48)	61.96 (51.65; 71.30)	93.48 (86.22; 97.04)^a^	73.91 (64.00; 81.87)	75.00 (65.16; 82.80)
Ultra-processed foods (total)	86.39 (83.54; 88.81)	53.54 (49.51; 57.34)	70.03 (66.40; 73.42)	91.59 (89.20; 93.49)	63.76 (60.00; 67.36)	65.44 (61.71; 69.00)
Soft drinks	88.89 (49.93; 98.47)^a^	11.11 (1.53; 50.08)	11.11 (1.53; 50.10)^b^ ^c^	88.89 (49.94; 98.47)^a^	11.11 (1.53; 50.06)^c^	11.11 (1.53; 50.06)^bc^
Sugar-sweetened beverages	73.98 (65.51; 80.98)^a^	17.89 (12.07; 25.69)	31.71 (24.09; 40.40)^b^ ^c^	83.74 (76.12; 89.27)^a^	34.15 (26.31; 42.96)^b^	22.76 (16.20; 31.01)^b^ ^c^
Dairy drinks	76.74 (61.90; 87.02)^a^	18.60 (9.57; 33.04)	34.88 (22.23; 50.10)	81.40 (66.97; 90.42)^a^	39.53 (26.17; 54.67)^a^	32.56 (20.31; 47.77)
Baked goods	96.77 (80.31; 99.55)	48.39 (31.64; 65.50)	80.65 (63.06; 91.00)	100.00^a^	64.52 (46.53; 79.16)	83.87 (66.60; 93.13)
Breakfast cereals	87.88 (71.79; 95.38)	87.88 (71.78; 95.38)	84.85 (68.34; 93.60)	100.00	87.88 (71.79; 95.38)	87.88 (71.79; 95.38)
Salty snacks	84.38 (67.49; 93.35)	66.67 (48.32; 81.06)	84.38 (67.49; 93.40)	90.63 (74.62; 96.95)	75.00 (57.38; 86.99)	84.38 (67.50; 93.35)
Candies	95.10 (90.08; 97.65)	70.99 (62.64; 78.13)	93.01 (87.48; 96.20)	99.30 (95.19; 99.90)	71.33 (63.38; 78.15)	76.92 (69.30; 83.11)
Cakes and pies	100.00	100.00	100.00	100.00	100.00	100.00
Dairy desserts	95.12 (82.45; 98.78)	78.95 (63.19; 89.12)	85.37 (71.02; 93.30)	100.00	73.17 (57.72; 84.49)	87.80 (73.83; 94.84)
Ultra-processed meat	68.42 (55.32; 79.13)	54.39 (41.43; 66.78)	64.91 (51.76; 76.10)	70.18 (57.13; 80.60)	68.42 (55.33; 79.13)	68.42 (55.33; 79.13)
Ready-to-eat food	95.24 (86.23; 98.46)	47.62 (35.65; 59.87)	80.95 (69.36; 88.90)	96.83 (88.15; 99.21)^a^	73.02 (60.78; 82.53)	79.37 (67.61; 87.63)
Sauces and creams	94.55 (84.38; 98.23)	80.49 (65.55; 89.94)	83.64 (71.42; 91.30)	94.55 (84.38; 98.23)	83.64 (71.42; 91.27)	85.45 (73.50; 92.56)
Total	87.10 (84.50; 89.32)	54.05 (50.25; 57.81)	69.28 (65.88; 72.48)	86.75 (84.21; 88.93)	65.87 (62.51; 69.08)	67.87 (64.55; 71.03)

Total sample size of foods and beverages with child-directed marketing = 1,054 products. In this table, we use all the nutrients of concern adopted in each NPM. ^a^ Statistically significant differences between Brazil NPM and other NPMs; ^b^ statistically significant differences between Mexico NPM and other NPMs; ^c^ statistically significant differences between PAHO NPM and other NPMs; differences between pairs were tested using the prevalence ratio and 95% CI.

Table 4 shows the proportion of foods with child-directed marketing and high in sodium across different NPMs. The Brazilian NPM allowed three times more foods and beverages to omit warnings for sodium (*p* < 0.05) than the PAHO NPM and 2.5 times more than the Mexican NPM. The Chilean and Uruguayan NPMs also allowed approximately two times more foods and beverages to omit warnings for sodium (*p* < 0.05) than the PAHO model. Analyzing the performance of the NPMs in labeling ultra-processed foods that are high in sodium, we found that the Brazilian NPM flagged three times fewer products than the PAHO and Mexican models, and the Uruguayan NPM labeled two times fewer ultra-processed foods than the PAHO model (*p* < 0.05). The Brazilian model also showed the lowest performance in labeling baked goods as high in sodium compared with all NPMs, except the Uruguayan NPM. The models from Uruguay and Chile allowed a significantly higher proportion of candies to omit warnings for sodium than the PAHO model. However, the Brazilian NPM identified a higher percentage of culinary ingredients high in sodium than the Mexican NPM (*p* < 0.05).

**Table 4:** Proportion (%) of foods and beverages with child-directed marketing and a high content of sodium according to different nutrient profile models (NPM), overall, and by food category (Brazil, 2017).

	**PAHO**	**Brazil**	**Chile**	**Mexico**	**Uruguay**	**Peru**
**Food groups**	**% (95% CI)**	**% (95% CI)**	**% (95% CI)**	**% (95% CI)**	**% (95% CI)**	**% (95% CI)**
Unprocessed and minimally processed foods	0.00	0.00	0.00	18.61 (14.10; 24.17)	5.63 (3.29; 9.45)	0.00
Culinary ingredients	0.00	11.76 (2.95; 36.89)^b^	4.26 (1.06; 15.51)	4.26 (1.06; 15.51)	4.26 (1.06; 15.51)	4.26 (1.06; 15.51)
Processed foods	79.35 (69.86; 86.43)	25.00 (10.79; 47.88)	52.13 (42.07; 62.02)	79.35 (69.86; 86.43)	44.57 (34.75; 54.82)	54.35 (44.11; 64.23)
Ultra-processed foods (total)	41.01 (37.29; 44.84)^a^	14.35 (11.80; 17.35)	24.00 (20.88; 27.43)	41.17 (37.44; 44.99)^a^	19.05 (16.21; 22.25)^c^	24.27 (21.12; 27.72)
Soft drinks	77.78 (42.04; 94.41)	0.00	0.00	77.78 (42.06; 94.41)	0.00	0.00
Sugar-sweetened beverages	39.02 (30.81; 47.92)	0.00	0.00	39.02 (30.81; 47.91)	0.00	0.00
Dairy drinks	18.60 (9.58; 33.03)	0.00	0.00	18.6 (9.58; 33.03)	0.00	2.33 (0.33; 14.79)^c^
Baked goods	87.1 (70.22; 95.08)^a^	12.9 (4.91; 29.78)	58.06 (40.40; 73.88)^a^	87.1 (70.23; 95.08)^a^	32.26 (18.30; 50.30)	58.06 (40.39; 73.88)^a^
Breakfast cereals	6.06 (1.52; 21.27)	0.00	3.03 (0.42; 18.65)	6.06 (1.52; 21.26)	0.00	3.03 (0.42; 18.66)
Salty snacks	56.25 (38.98; 72.13)	40 (24.28; 58.09)	69.7 (52.24; 82.87)	56.25 (38.99; 72.12)	50.00 (33.32; 66.68)	68.75 (51.00; 82.30)
Candies	14.79 (9.84; 21.63)	0.00	2.1 (0.68; 6.31)^c^	14.79 (9.84; 21.63)	1.41 (0.35; 5.46)^c^	2.11 (0.68; 6.35)^c^
Cakes and pies	0.00	0.00	0.00	0.00	0.00	0.00
Dairy desserts	7.32 (2.37; 20.40)	0.00	0.00	7.32 (2.38; 20.39)	0.00	2.44 (0.34; 15.43)
Ultra-processed meat	67.86 (54.63; 78.73)	50.00 (37.17; 62.83)	64.29 (51.01; 75.68)	69.64 (56.47; 80.23)	57.14 (43.98; 69.37)	64.29 (51.01; 75.68)
Ready-to-eat food	80.65 (68.91; 88.68)^a^	27.42 (17.76; 39.78)	59.68 (47.10; 71.10)	80.65 (68.92; 88.67)^a^	48.39 (36.28; 60.69)	59.68 (47.10; 71.10)
Sauces and creams	81.82 (69.38; 89.94)	68.29 (52.70; 80.63)	69.64 (56.47; 80.23)	81.82 (69.38; 89.94)	61.82 (48.43; 73.62)	70.91 (57.64; 81.36)
Porridge flour	0.00	0.00	0.00	0.00	0.00	0.00
Total	42.96 (39.56; 46.47)^a^	14.48 (12.00; 17.37)	20.43 (18.11; 22.96)^c^	36.35 (33.50; 39.29)^a^	16.94 (14.81; 19.33)^c^	26.38 (23.43; 29.56)

Total sample size of foods and beverages with child-directed marketing = 1,054 products; ^a^statistically significant differences between Brazil NPM and other NPMs; ^b^statistically significant differences between Mexico NPM and other NPMs.; ^b^statistically significant differences between PAHO NPM and other NPMs; differences between pairs were tested using the prevalence ratio and 95% CI.

Table 5 shows the proportion of foods with child-directed marketing and high in saturated fat across different NPMs. The Chilean NPM allowed 2.2 times more foods and beverages to omit warnings for saturated fat (*p* < 0.05) than the PAHO NPM. The Brazilian NPM allowed up to 15 times more processed foods to omit warnings for saturated fat (*p* < 0.05) than other models. Also, the Brazilian NPM allowed approximately five times more cakes and pies to omit warnings for saturated fat (*p* < 0.05) than PAHO, Mexican, Chilean, and Peru models. The models from Uruguay and Peru labeled eight times more processed foods high in saturated fat than the Mexican model (*p* < 0.05).

**Table 5:** Proportion (%) of foods and beverages with child-directed marketing and a high content of saturated fat according to different nutrient profile models (NPM), overall, and by food category (Brazil, 2017).

	**PAHO**	**Brazil**	**Chile**	**Mexico**	**Uruguay**	**Peru**
**Food groups**	**% (95% CI)**	**% (95% CI)**	**% (95% CI)**	**% (95% CI)**	**% (95% CI)**	**% (95% CI)**
Unprocessed and minimally processed foods	0.00	0.00	0.00	0.00	0.00	0.00
Culinary ingredients	0.00	52.94 (30.23; 74.50)	4.17 (1.04; 15.21)	8.33 (3.16; 20.21)	72.92 (58.74; 83.59)^b^	72.92 (58.73; 83.59) ^b^
Processed foods	77.17 (67.49; 84.63)^a^	5.00 (0.70; 28.30)	13.04 (7.55; 21.59)^ac^	78.26 (68.67; 85.53)^a^	60.87 (50.57; 70.29)^a^	67.39 (57.18; 76.18)^a^
Ultra-processed foods (total)	36.77 (33.14; 40.55)	20.48 (17.48; 23.85)	25.38 (22.18; 28.88)	37.08 (33.44; 40.86)	22.61 (19.56; 25.99)	31.23 (27.78; 34.90)
Soft drinks	0.00	0.00	0.00	0.00	0.00	0.00
Sugar-sweetened beverages	0.81 (0.11; 5.56)	0.00	0.00	0.81 (0.11; 5.55)	0.00	0.00
Dairy drinks	32.56 (20.31; 47.77)	0.00	0.00	34.88 (22.24; 50.09)	0.00	2.33 (0.33; 14.79)^c^
Baked goods	16.13 (6.87; 33.40)	3.23 (0.45; 19.70)	3.23 (0.45; 19.68)	16.13 (6.87; 33.39)	3.23 (0.45; 19.68)	9.68 (3.15; 26.09)
Breakfast cereals	36.36 (21.93; 53.76)	30.3 (17.13; 47.77)	39.39 (24.42; 56.67)	36.36 (21.93; 53.75)	30.30 (17.13; 47.76)	45.45 (29.55; 62.34)
Salty snacks	45.16 (28.85; 62.58)	44.83 (28.07; 62.84)	48.39 (31.65; 65.49)	45.16 (28.86; 62.57)	41.94 (26.12; 59.60)	48.39 (31.65; 65.50)
Candies	45.77 (37.75; 54.02)	35.38 (27.64; 43.98)	49.3 (41.16; 57.47)	45.77 (37.76; 54.02)	36.62 (29.10; 44.85)	50.00 (41.84; 58.16)
Cakes and pies	62.5 (37.69; 82.12)^a^	12.5 (3.14; 38.66)	68.75 (43.29; 86.38)^a^	62.50 (37.70; 82.11 a)	12.50 (3.14; 38.64)^c^	68.75 (43.28; 86.38)^a^
Dairy desserts	47.5 (32.70; 62.75)	18.42 (9.03; 33.93)	12.5 (5.29; 26.74)^c^	47.50 (32.71; 62.74)	17.50 (8.57; 32.43)	35.00 (21.93; 50.79)
Ultra-processed meat	60.71 (47.46; 72.56)	39.29 (27.44; 52.55)	17.86 (9.88; 30.12)^c^	62.50 (49.23; 74.12)	39.29 (27.44; 52.54)	51.79 (38.85; 64.48)
Ready-to-eat food	47.62 (35.65; 59.87)	19.05 (11.14; 30.64)	20.63 (12.37; 32.38)	47.62 (35.66; 59.86)	19.05 (11.14; 30.63)	25.40 (16.17; 37.53)
Sauces and creams	61.82 (48.43; 73.63)	34.15 (21.36; 49.75)	49.09 (36.22; 62.08)	61.82 (48.43; 73.62)	50.91 (37.92; 63.78)	50.91 (37.92; 63.78)
**Total**	40.33 (36.96; 43.78)	20.66 (17.74; 23.92)	17.73 (15.59; 20.16)^c^	34.72 (31.90; 37.64)	23.49 (21.03; 26.14)	37.69 (34.38; 41.15)

Notes: Total sample size of foods and beverages with child-directed marketing = 1,054 products; ^a^ statistically significant differences between Brazil NPM and other NPMs; ^b^ statistically significant differences between Mexico NPM and other NPMs.; ^c^ statistically significant differences between PAHO NPM and other NPMs; differences between pairs were tested using prevalence ratio and 95% CI.

Table 6 shows the proportion of foods with child-directed marketing and high in free sugar by applying different NPMs. The Brazilian NPM allows around four times more sugar-sweetened beverages and six times more dairy drinks to omit warnings for free sugar (*p* < 0.05) than the PAHO and Mexican NPMs. The Peru NPM allows around three times more sugar-sweetened beverages to omit warnings for free sugar (*p* < 0.05) than the Mexican model.

**Table 6:** Proportion (%) of foods and beverages with child-directed marketing and high content of free sugar according to different nutrient profile models (NPMs), overall, and by food category (Brazil, 2017).

	**PAHO**	**Brazil**	**Chile**	**Mexico**	**Uruguay**	**Peru**
**Food groups**	**% (95% CI)**	**% (95% CI)**	**% (95% CI)**	**% (95% CI)**	**% (95% CI)**	**% (95% CI)**
Unprocessed and minimally processed foods	0.00	0.00	0.00	0.00	0.00	0.00
Culinary ingredients	0.00	29.41 (12.78; 54.23)	0.00	10.42 (4.40; 22.72)	12.50 (5.72; 25.18)	10.42 (4.40; 22.72)
Processed foods	7.61 (3.67; 15.13)	5.00 (0.69; 28.30)	6.52 (2.95; 13.78)	13.04 (7.55; 21.59)	5.43 (2.28; 12.41)	6.52 (2.96; 13.78)
Ultra-processed foods (total)	56.27 (52.43; 60.00)	38.36 (34.62; 42.25)	48.93 (45.11; 52.77)	57.64 (53.81; 61.39)	42.97 (39.21; 46.80)	43.12 (39.36; 46.95)
Soft drinks	11.11 (1.53; 50.07)	11.11 (1.53; 50.08)	11.11 (1.53; 50.07)	11.11 (1.53; 50.06)	11.11 (1.53; 50.06)	11.11 (1.53; 50.06)
Sugar-sweetened beverages	51.22 (42.43; 59.94)^a^	17.89 (12.07; 25.69)	31.71 (24.09; 40.45)	55.28 (46.41; 63.83)^a^	32.52 (28.43; 41.29)	22.76 (16.20; 31.01)^b^
Dairy drinks	72.09 (56.99; 83.43)^a^	18.60 (9.57; 33.04)	34.88 (22.23; 50.10)	81.40 (66.97; 90.42)^a^	30.23 (18.42; 45.40)	32.56 (20.31; 47.77)
Baked goods	58.06 (40.39; 73.88)	41.94 (26.11; 59.61)	48.39 (31.64; 65.50)	58.06 (40.39; 73.88)	41.94 (26.12; 59.61)	48.39 (31.65; 65.50)
Breakfast cereals	84.85 (68.34; 93.56)	84.85 (68.34; 93.56)	84.85 (68.34; 93.56)	84.85 (68.35; 93.56)	84.85 (68.35; 93.56)	84.85 (68.35; 93.56)
Salty snacks	46.88 (30.56; 63.88)	33.33 (18.94; 51.68)	43.75 (27.87; 61.02)	46.87 (30.57; 63.88)	34.38 (20.15; 52.10)	43.75 (27.87; 61.02)
Candies	84.64 (77.73; 89.66)	69.47 (61.05; 76.75)	87.41 (80.89; 91.93)	84.62 (77.73; 89.66)	68.53 (60.46; 75.62)	70.63 (62.65; 77.52)
Cakes and pies	100.00	100.00	100.00	100.00	100.00	100.00
Dairy desserts	90.24 (76.70; 96.30)	68.42 (5.20; 81.13)	82.93 (68.25; 91.65)	90.24 (76.70; 96.30)	68.29 (52.71; 80.63)	82.93 (68.26; 91.65)
Ultra-processed meat	0.00	0.00	0.00	0.00	0.00	0.00
Ready-to-eat food	36.51 (25.60; 49.01)	19.05 (11.14; 30.64)	31.75 (21.47; 44.17)	36.51 (25.60; 49.01)	26.98 (17.47; 39.22)	28.57 (18.79; 40.88)
Sauces and creams	18.18 (10.06; 30.62)	17.07 (8.35; 31.75)	14.55 (7.44; 26.50)	18.18 (10.06; 30.62)	20.00 (11.43; 32.64)	14.55 (7.44; 26.50)
**Total**	50.66 (47.09; 54.23)	37.69 (34.08; 41.44)	44.15 (40.63; 47.72)	50.00 (46.53; 53.46)	37.25 (33.96; 40.66)	37.37 (34.08; 40.79)

Total sample size of foods and beverages with child-directed marketing = 1,054 products; ^1^statistically significant differences between Brazil NPM and other NPMs; ^b^statistically significant differences between Mexico NPM and other NPMs.

## Discussion

This study applied different NPMs from LA to a sample of 1,054 foods and beverages with the presence of child-directed marketing sold in Brazil. Sugar-sweetened beverages had the highest prevalence of child-directed marketing strategies. A higher content of free sugars (g), saturated fat (g), and sodium (mg) per 100 kcal was observed among subgroups of ultra-processed foods including candies, cakes and pies, sauces and creams, and sugar sweetened-beverages with child-directed marketing when compared with the same food groups without child-directed marketing. In addition, the proportion and types of foods and beverages with child-directed marketing high in nutrients of concern varied across NPMs. The PAHO and Mexican NPMs were the most effective profiling schemes in labeling foods high in nutrients of concern with the presence of child-directed marketing among all. The differences observed were more remarkable when comparing these two models with the Brazilian model, which allowed more foods targeted at children, especially ultra-processed foods, to omit warning labels for free sugar, sodium, and saturated fat. The models of Chile, Uruguay, and Peru would also target significantly fewer sugar-sweetened beverages with child-directed marketing and high in at least one critical nutrient than PAHO and Mexican ones.

Previous studies conducted in Brazil (39), Canada (58), Mexico (40), Peru (59), England (60, 61), Chile (31), South Africa (37), and Australia (41) have shown that the choice of the NPM can have a substantial impact on different food policies. The NPM adopted by governments can affect the proportion of foods and beverages that receive an FoP warning label for nutrients linked to an increased risk of NCD and also could be used as a criterion to define foods and beverages subject to marketing and sales restrictions in all types of media and in schools' food environment (30, 31, 62). Regarding the use of the NPM for regulations on unhealthy food marketing to children, a study showed that 16 countries have adopted regulations addressing this issue, and in 10 of these countries, the NPM was used to identify which products should qualify for restrictions (30). Currently, four countries apply an NPM to all foods and beverages that exceed critical values for the nutrients included in the model (United Kingdom, Ireland [except cheese products], Taiwan, and Chile), while six countries apply an NPM for specific food and beverage categories (South Korea, Mexico, Ecuador, Poland, Uruguay, and Turkey) (30). Unlike the policies implemented in Chile (38) and Mexico (40), the Brazilian NPM will not be used to restrict child-directed marketing on food packages as part of its public health policy.

The nutrient profile models proposed in South Africa were adapted from the Chilean NPM and found to be suitable for food labeling regulation in that context (37). However, our results showed that the Chilean model compared with the PAHO and Mexican models was not effective in identifying foods and beverages with the presence of child-direct marketing as high in sugar. The Chilean model overall also identifies lower proportions of foods and beverages targeted at children and high in sodium and saturated fat than the PAHO model. However, the implementation of the NPM in Chile has led to a decline in the proportion of advertisements for foods high in nutrients of concern, especially soft drinks, desserts, breakfast cereals, and fruit-flavored drinks (33). This may be partly because of the NPM cutoff point and eligibility criteria adopted but could also be attributed to a range of other regulatory measures (e.g., taxes) implemented in that context (28).

We found that ultra-processed foods and beverages with child-directed marketing such as candies, cakes and pies, sauces and creams, and sugar-sweetened beverages had a higher content of free sugar, saturated fat, and sodium per 100 kcal than those same food items without child-directed marketing. A great proportion of these products would not receive warning labels for high sodium, saturated fat, and sugar by NPMs from Brazil, Uruguay, Chile, and Peru. Previous studies have also found poorer nutrient compositions for products with child-directed marketing in Brazil and Canada (58, 63). Studies conducted in Mexico (40), Brazil (63), Colombia (64), and Peru (59) found that packaged foods with child-directed marketing are often high in sugar, fat, and sodium and low in fiber. By applying the Peruvian NPM, ~88% of breakfast cereals with the presence of child-directed marketing would receive the FoP nutritional labeling for being high in at least one nutrient of concern. However, compared with stronger models (PAHO and Mexico), like the Peruvian NPM, a significantly lower proportion of carbonated soft drinks and sugar-sweetened beverages targeted at children would be eligible to receive warning labels for at least one critical nutrient. Corroborating our findings, another study showed significant discrepancies between the PAHO and Brazilian NPMs in labeling products with child-directed marketing and high levels of critical nutrients, especially for fruit drinks, dairy beverages, sandwich cookies, cakes, breakfast cereals, jellies, corn snacks, and yogurts (65).

In our study, the most effective models for labeling sugar-sweetened beverages high in free sugar and/or sodium targeted at children were the Mexican and PAHO models not only because the cutoff points of their nutrient profile models were higher than the others but also because the eligibility criteria that take the food processing dimension into account, as is the case with the PAHO model (35). The NPMs adopted in LA were less likely to identify foods high in sugar, saturated fat, and sodium or high in at least one nutrient of concern with the presence of child-directed marketing than the PAHO and Mexican NPMs. Our study highlights the need to review the thresholds of the Brazilian NPM for free sugar, saturated fat, and sodium and the eligibility criteria, especially for ultra-processed foods and beverages, should the Brazilian government intend to regulate child-directed marketing on packages. SSBs and other ultra-processed beverages with the presence of child-directed marketing on the package were less likely to be targeted by the Brazilian NPM than the other NPMs adopted in LA, and these products are frequently consumed by Brazilian children and are related to an increase in the risk for obesity, type 2 diabetes mellitus, and cardiovascular diseases (9, 63).

This study is not free of limitations. First, the subsample of products used in the analyses may not represent children's food intake in Brazil; however, several groups of ultra-processed foods associated with obesity and other NCDs in this age group were considered, such as carbonated soft drinks, sugar-sweetened beverages, dairy beverages, candies, and salty snacks (7–10, 59). Second, the sample might not be representative of the wider universe of products with marketing. Third, the estimated proportion of foods and beverages with child-directed marketing may have been misclassified, given our difficulty to discriminate marketing strategies geared toward children from other types of marketing strategies (50). Finally, because the Brazilian legislation does not require foods and beverages sold in the country to display the content of free sugars on the nutrition facts panel, our estimates for free sugar may be biased (57). The sensitivity analyses conducted however did not show the direction of the error since, for some food groups, the estimate increased the amount of free sugar per 100 g and in others decreased it compared to that reported on the package (Supplementary Table 3). We tested the inter-rater agreement for free sugar values estimated by using this method for two senior researchers independently and found a 90% of agreement (kappa value = 0.75) (data not shown).

Strengths of our study include the use of a sample of foods from the largest Brazilian food retailers, the careful characterization of food packages with child-directed marketing using internationally recommended protocols for monitoring public food policies, and the comparison between NPMs adopted in Latin American countries. Our findings provide evidence of how the choice of NPM is a key part of food policy design and planning when the policy target is the promotion of healthy eating and the fight against childhood obesity.

In conclusion, we identified greater proportions of ultra-processed foods with child-directed marketing high in sugar, sodium, and/or saturated fat using the NPM suggested by PAHO and adopted in Mexico were better than the regulations adopted in Brazil to identify which foods should receive FOP nutrition labeling. Our findings suggest that the NPM criteria adopted in Brazil are also limited to support marketing restriction policies to protect children.

## Data availability statement

The raw data supporting the conclusions of this article will be made available by the authors, without undue reservation.

## Author contributions

CB contributed to the design of the study and leaded the data analysis and writing of this manuscript. NK contributed to the writing of this manuscript and reviewed the data analysis. DN contributed to the writing of this manuscript. AD contributed to the design of the study and reviewed the data analysis. All authors read and approved the final manuscript.

## Funding

This research was funded by Bloomberg Philanthropies through a sub award agreement between the University of North Carolina at Chapel Hill and the Medicine Faculty Foundation (FFM), grant number 5104695. The funder has no role in the study design, data collection and analysis, decision to publish, or preparation of the manuscript.

## Conflict of interest

The authors declare that the research was conducted in the absence of any commercial or financial relationships that could be construed as a potential conflict of interest.

## Publisher's note

All claims expressed in this article are solely those of the authors and do not necessarily represent those of their affiliated organizations, or those of the publisher, the editors and the reviewers. Any product that may be evaluated in this article, or claim that may be made by its manufacturer, is not guaranteed or endorsed by the publisher.
