# Supplementary material for: Comparing Latin American nutrient profile models using data from packaged foods with child-directed marketing within the Brazilian food supply
Source: Front Nutr. 2022 Dec 2;9:920710. doi: 10.3389/fnut.2022.920710 (PMC9755586; doi:10.3389/fnut.2022.920710)
Supplement: Supplementary file 1 [file Table_1.docx]

***Supplementary Material***

# Supplementary Tables

- 1. Food and beverage groups and subgroups analyzed in the 5 main food retailers in Brazil.

|  | |
| --- | --- |
| **Food groups** | **Foods subgroups** |
| Non-dairy beverages | |
| Carbonated beverages | Carbonated beverages, including artificially sweetened versions. |
| Fruit juices | Products declared as juices, fruit juices without water or added sugar, coconut water. |
| Fruit-flavored drinks | Fruit drink powder, fruit punch concentrate, fruit-flavored beverages. |
| Nectars | Nectars made with juice and added water and/or sugar. |
| Coffee and tea | Coffee grains and powder, tea sachets and leaves. |
| Other beverages | Plant-based beverages, ready-to-drink teas, isotonic drinks, coconut milk. |
| Dairy | |
| Sweetened dairy beverages | Sweetened yogurt, flavored milks, fermented milk, milk compounds, dairy beverages. |
| Unsweetened dairy beverages | Unsweetened yogurt, milks, evaporated and powdered milk, milk compounds. |
| Cheese and cheese spreads | Processed and ultra-processed cheese, cheese spreads. |
| Salty snacks | Salted peanuts, salty snacks, potato chips, potato sticks, microwave popcorn. |
| Cookies and crackers | Sweet and savory crackers, biscuits, and cookies. |
| Sweets, desserts, and convenience foods | |
| Candies and desserts | Chocolate bars, candies, condensed milk, cocoa powder and sweetened dairy mixes, jellies, syrups, chocolate spreads, chewing gum, marshmallows, ice cream (including versions with nonnutritive sweeteners). |
| Fruit preserve | Fruit jellies, fruit preserves, canned fruits, dried fruits, fruit sorbets. |
| Convenience foods | Ready-to-eat meals, frozen french fries, instant rice, instant noodles, instant soups, instant mashed potatoes, stuffed pasta, frozen pizzas, frozen and ready-to-eat pies, sandwiches, baby foods. |
| Processed meats | Burgers, sausages, canned fish, smoked meats, seasoned meats, salted meats, hams, salami, spreads |
| Sauces, herbs, and dressings | Sauces, mayonnaises, herbs, catchup, salad dressings. |
| Bakery products | Breads, toasts, and cakes (including powders). |
| Breakfast cereals and granola bars | Corn flakes, flavored oats, infant cereals, granolas, mueslis, granola bars, porridges, mix of cereals and fruits. |
| Culinary ingredients | |
| Sugar and other nonnutritive sweeteners | Sugar, honey, nonnutritive sweeteners. |
| Oils and fats | Oils, margarines, butters, fresh cream, fats. |
| Other minimally processed and processed foods | |
| Canned vegetables | Canned beans and vegetables. |
| Cereals, beans, other grain products | Dry beans, flours, rice, corn, pasta. |
| Meat, poultry, seafood, and egg | Meat, poultry, seafood, egg, including chilled and frozen products. |
| Nuts and seeds | Nuts and seeds, including salted nuts. |
| Packaged fruits and vegetables | Fresh and frozen fruits and vegetables, frozen fruit pulp. |

## Assessed health/nutrition claims and marketing strategies on food label according to INFORMAS taxonomy and categorization of child-directed marketing from this taxonomy

| INFORMAS Taxonomy | INFORMAS components | Components/terms present on food label that have been classified as child-directed marketing* |
| --- | --- | --- |
| Promotional Characters | 1. Cartoon/Company-owned character, e.g. M&Ms2. Licenced character, e.g. Dora the explorer3. Amateur sportsperson, e.g. person playing a sport4. Famous sportsperson/team, e.g. All Blacks (Rugby team NZ)5. Celebrity/famous (non-sports) figure, e.g. Jamie Oliver6. Movie tie-in, e.g. Shreck7. Non-sports/historical events/festivals, e.g. Christmas, ANZAC day8. ‘For kids’ e.g. image of a child, e.g. ‘great for school lunches’9. Awards, e.g. Best Food Award 2014, award winning10. Sports event, e.g. Rugby World Cup | 1. Cartoon/Company-owned character, e.g. M&Ms2. Licenced character, e.g. Dora the explorer3. Amateur sportsperson, e.g. person playing a sport4. Famous sportsperson/team, e.g. All Blacks (Rugby team NZ)6. Movie tie-in, e.g. Shreck7. Non-sports/historical events/festivals, e.g. Christmas, ANZAC day8. ‘For kids’ e.g. image of a child, e.g. ‘great for school lunches’10. Sports event, e.g. Rugby World Cup |
| Premium Offers | 1= Game and app downloads2= Contests3= Pay 2 take 3 or other4= 20% extra or other5= Limited edition6= Social charity7= Gift or collectable8= Price discount9= Loyalty programs | 1= Game and app downloads7= Gift or collectable |
| Nutrient Claims | *a) Health-related ingredient claims**b) Nutrient content claims**c) Nutrient comparative claims*To review all the examples of terms that are considered nutrient claims see the link: https://www.informas.org/modules/food-labelling/ | Terms used:“Made from 50% real fruit”“Superfruits”“30% more apricots” |
| Health Claims | *a) Nutrient and other function claims**b) Reduction of disease risk claims**c) General health claims*To review all the examples of terms that are considered health claims see the link: https://www.informas.org/modules/food-labelling/ | Terms used:"super-healthy",“superfood”"energetic or that gives you energy","power energy","growth or development""health bones"“for growing kids”“nutrient and growth e.g. protein for development” |

## * The choice of terms was based on the references listed below:

## Cairns G, Angus K, Hastings G, Caraher M. Systematic reviews of the evidence on the nature, extent and effects of food marketing to children. A retrospective summary. Appetite. 2013;62:209–15.

## Cruz-Casarrubias C, Tolentino-Mayo L, Nieto C,Théodore FL, Monterrubio-Flores E. Use of advertising strategies to target children in sugar-sweetened beverages packaging in Mexico and the nutritional quality of those beverages. *Pediatr. Obes.* 2020, e12710

## Elliott C. Assessing “fun foods”: nutritional content and analysis of supermarket foods targeted at children. Obes Rev Off J Int Assoc Study Obes. 2008;9(4):368–77.

## Elliott C, Truman E. The Power of Packaging: A Scoping Review and Assessment of Child-Targeted Food Packaging. Nutrients. 2020;12(4):958.

## Gilbert-Moreau J, Pomerleau S, Perron J, Gagnon P, Labonté M-È, Provencher V. Nutritional value of child-targeted food products: results from the Food Quality Observatory. Public Health Nutr. 2021;24(16):5329–37.

## Perry A, Chacon V, Barnoya J. Health claims and product endorsements on child-oriented beverages in Guatemala. Public Health Nutr. 2018;21(3):627–31.

## Sadeghirad B, Duhaney T, Motaghipisheh S, Campbell NRC, Johnston BC. Influence of unhealthy food and beverage marketing on children’s dietary intake and preference: a systematic review and meta-analysis of randomized trials. Obes Rev Off J Int Assoc Study Obes. 2016;17(10):945–59.

## Taillie LS, Busey E, Stoltze FM, Dillman Carpentier FR. Governmental policies to reduce unhealthy food marketing to children. Nutr Rev. 2019;77(11):787–816.

- 1. Sensitivity analysis for free sugar.
- Mean of total sugar in 100g of the product found on the nutrition fact panel at label of the food and beverage collected in the 5 main food retailers in Brazil.

|  | **n** | **Mean** | **95 % CI** |
| --- | --- | --- | --- |
| soft drinks | 51 | 4.92 | 3.52; 6.32 |
| sugar-sweetened beverages | 258 | 16.82 | 13.80; 19.84 |
| dairy drinks | 52 | 9.32 | 6.32; 12.32 |
| baked goods | 20 | 1.78 | 0.55; 3.00 |
| breakfast cereals | 91 | 22.70 | 19.87; 25.52 |
| salty snacks | 92 | 4.45 | 3.23; 5.66 |
| candies | 306 | 16.06 | 3.44; 28.67 |
| cakes and pies | 14 | 20.27 | 17.98; 22.57 |
| dairy desserts | 71 | 7.60 | 4.65; 10.54 |
| ultra-processed meats | 0 | 0.00 | . |
| ready-to-eat food | 35 | 7.32 | 5.41; 9.24 |
| sauces and creams | 80 | 33.00 | . |

- Mean of free sugar in 100g of the product estimated by the 8-step methodology according to Scapin et al.

|  | **N** | **mean** | **95% CI** |
| --- | --- | --- | --- |
| soft drinks | 79 | 6,89 | 5,98; 7,80 |
| sugar-sweetened beverages | 616 | 9,92 | 8,49; 11,36 |
| dairy drinks | 275 | 10,16 | 8,66; 11,66 |
| baked goods | 296 | 15,75 | 13,54; 17,96 |
| breakfast cereals | 258 | 36,15 | 34,13; 38,17 |
| salty snacks | 531 | 27,69 | 25,51; 29,87 |
| candies | 1,266 | 42,41 | 41,15; 43,67 |
| cakes and pies | 250 | 34,04 | 32,67; 35,41 |
| dairy desserts | 464 | 31,53 | 29,63; 33,43 |
| ultra-processed meats | 412 | 1,01 | 0,84; 1,19 |
| ready-to-eat food | 596 | 9,4 | 8,15; 10,65 |
| sauces and creams | 624 | 5,44 | 4,59; 6,31 |
